# Frequencies distribution of dihydrofolate reductase and dihydropteroate synthetase mutant alleles associated with sulfadoxine–pyrimethamine resistance in *Plasmodium falciparum* population from Hadhramout Governorate, Yemen

**DOI:** 10.1186/s12936-015-1035-2

**Published:** 2015-12-22

**Authors:** Omar A. A. Bamaga, Mohammed A. K. Mahdy, Yvonne A. L. Lim

**Affiliations:** Department of Parasitology, Faculty of Medicine, University of Malaya, 50603 Kuala Lumpur, Malaysia; Department of Parasitology, Faculty of Medicine, Sana’a University, Sana’a, Yemen; Tropical Disease Research Centre, University of Science and Technology, Taiz, Yemen

**Keywords:** *Plasmodium falciparum*, *Pfdhfr*, *Pfdhps*, Sulphadoxine–pyrimethamine resistance, Molecular marker, Hadhramout-Yemen

## Abstract

**Background:**

Malaria in Yemen is mainly caused by *Plasmodium falciparum* and 25 % of the population is at high risk. Sulfadoxine–pyrimethamine (SP) had been used as monotherapy against *P. falciparum*. Emergence of chloroquine resistance led to the shift in anti-malarial treatment policy in Yemen to artemisinin-based combination therapy, that is artesunate (AS) plus SP as first-line therapy for uncomplicated malaria and artemether–lumefantrine as second-line treatment. This study aimed to screen mutations in the dihydrofolate reductase (*dhfr*) and dihydropteroate synthetase (*dhps*) genes associated with SP resistance among *P. falciparum* population in Hadhramout governorate, Yemen.

**Methods:**

Genomic DNA was extracted from dried blood spots of 137 *P. falciparum* isolates collected from a community-based study. DNA was amplified using nested polymerase chain reaction (PCR) and subsequently sequenced for *Pfdhfr* and *Pfdhps* genes. Sequences were analysed for mutations in *Pfdhfr* gene codons 51, 59, 108, and 164 and in *Pfdhps* gene codons 436, 437, and 540.

**Results:**

A total of 128 and 114 *P. falciparum* isolates were successfully sequenced for *Pfdhfr* and *Pfdhps* genes, respectively. Each *Pfdhfr* mutant allele (**I**_**51**_ and **N**_**108**_) in *P. falciparum* population had a frequency of 84 %. *Pfdhfr***R**_**59**_ mutant allele was detected in one isolate. Mutation at codon 437 (**G**_**437**_) in the *Pfdhps* gene was detected in 44.7 % of *falciparum* malaria isolates. Frequencies of *Pfdhfr* double mutant genotype (**I**_**51**_C_59_**N**_**108**_I_164_) and *Pfdhfr*/*Pfdhps* triple mutant genotype (**I**_**51**_C_59_**N**_108_I_164_-S_436_**G**_437_K_540_) were 82.8 and 39.3 %, respectively. One isolate harboured *Pfdhfr* triple mutant genotype (**I**_**51**_, **R**_**59**_, **N**_**108**_, I_164_) and *Pfdhfr*/*Pfdhps* quadruple mutant genotype (**I**_51_**R**_59_**N**_108_I_164_-S_436_**G**_437_K_540_).

**Conclusion:**

High frequencies of *Pfdhfr* and *Pfdhps* mutant alleles and genotypes in *P. falciparum* population in Hadhramout, Yemen, highlight the risk of developing resistance for SP, the partner drug of AS, which subsequently will expose the parasite to AS monotherapy increasing then the potential of the emergence of AS resistance. Study findings necessitate the continuous monitoring of the efficacy of the national anti-malarial drugs policy in Yemen. In addition, monitoring SP efficacy using molecular markers that has shown to be a practical and informative method for monitoring the partner drug of AS.

## Background

Malaria is a major health problem in Yemen, where more than 25 % of the population are at considerably high risk of malaria with 149,451 confirmed cases in 2013 [1]. Malaria in Yemen belongs to the afro-tropical type with the predominance of *Plasmodium falciparum* and *Anopheles arabiensis* as the predominant vector. However, malaria epidemiology in Socotra Island and the eastern governorate of Al-Maharah belongs to the oriental type with *Anopheles culicifacies* as the predominant vector [2, 3]. The National Malaria Control Programme (NMCP) in Yemen is proactive in controlling malaria through prompt diagnosis and proper treatment, distribution of insecticide-treated mosquito nets, indoor residual spraying, and active case surveillance [4].

The national anti-malarial drug policy in Yemen was formulated in 1999, including chloroquine (CQ) as first-line and sulfadoxine–pyrimethamine (SP) as a second line monotherapy for treating uncomplicated falciparum malaria [5]. In 2005, due to the increased CQ resistance, anti-malarial drug policy shifted to a combination of artesunate (AS) and SP as the first-line therapy and artemether–lumefantrine as a second-line treatment for uncomplicated malaria [6]. Continued use of SP in the new policy, availability of this drug in the private sector, and poor knowledge of the national policy among physicians [7] may increase the monotherapy of SP against *P. falciparum*, which is likely to compromise drug efficacy. Monitoring anti-malarial drug efficacy in Yemen started in 2002 following the WHO protocol for in vivo assessment in four sentinel sites. In 2004, three in vivo studies on the efficacy of SP showed success rate ranging from 95 to 100 %. Four years later, after launching the new policy, in vivo efficacy trails conducted in three monitoring sites reported 97.6–100 % adequate clinical and parasitological response (ACPR) for AS + SP [3]. The efficacity of AS + SP as first-line treatment for uncomplicated falciparum malaria was also rated at 97 % ACPR in a recent clinical drug efficacy trail carried out in 2013 [8]. It is noteworthy that currently used routine clinical efficacy trail is the gold standard for the assessment of the efficiency of the combined anti-malarial drugs, although it does not differentiate between the efficacy of AS and its partner drug.

Molecular markers are practical for monitoring SP resistance. Quintuple mutant of combined dihydrofolate reductase (*dhfr*) and dihydropteroate synthase (*dhps*) genes (*Pfdhfr***I**_**51**_, **R**_**59**_, **N**_**108**_ plus *Pfdhps***G**_**437**_, **E**_**540**_) was significantly associated with in vivo resistance to SP [9]. In Yemen, mutant allele **R**_**59**_ of *pfdhfr* was detected in 5 % of *P. falciparum* isolates (5/99) in Lahj governorate, southern Yemen [10]. Double mutant genotype of *pfdhfr* (**I**_**51**_/**N**_**108**_) was reported in 54 % of *P. falciparum* isolates in Taiz, Dhamar, and Hodeidah governorates in western Yemen [11]. *Pfdhfr* mutant allele (**N**_**108**_) was also reported in 53.2 % of *P. falciparum* isolates collected from Hodeidah governorate [12]. However, data on *Pfdhfr* and *Pfdhps* mutant alleles and genotypes are not available from the southeastern governorates of Yemen. This study aimed to screen *Pfdhfr* and *Pfdhps* mutant alleles and genotypes among *P. falciparum* population isolated from a community-based survey conducted in Hadhramout. Findings from this study will be used to predict the development of SP resistance.

## Methods

### Study sites/subjects and sampling

**Fig. 1:**
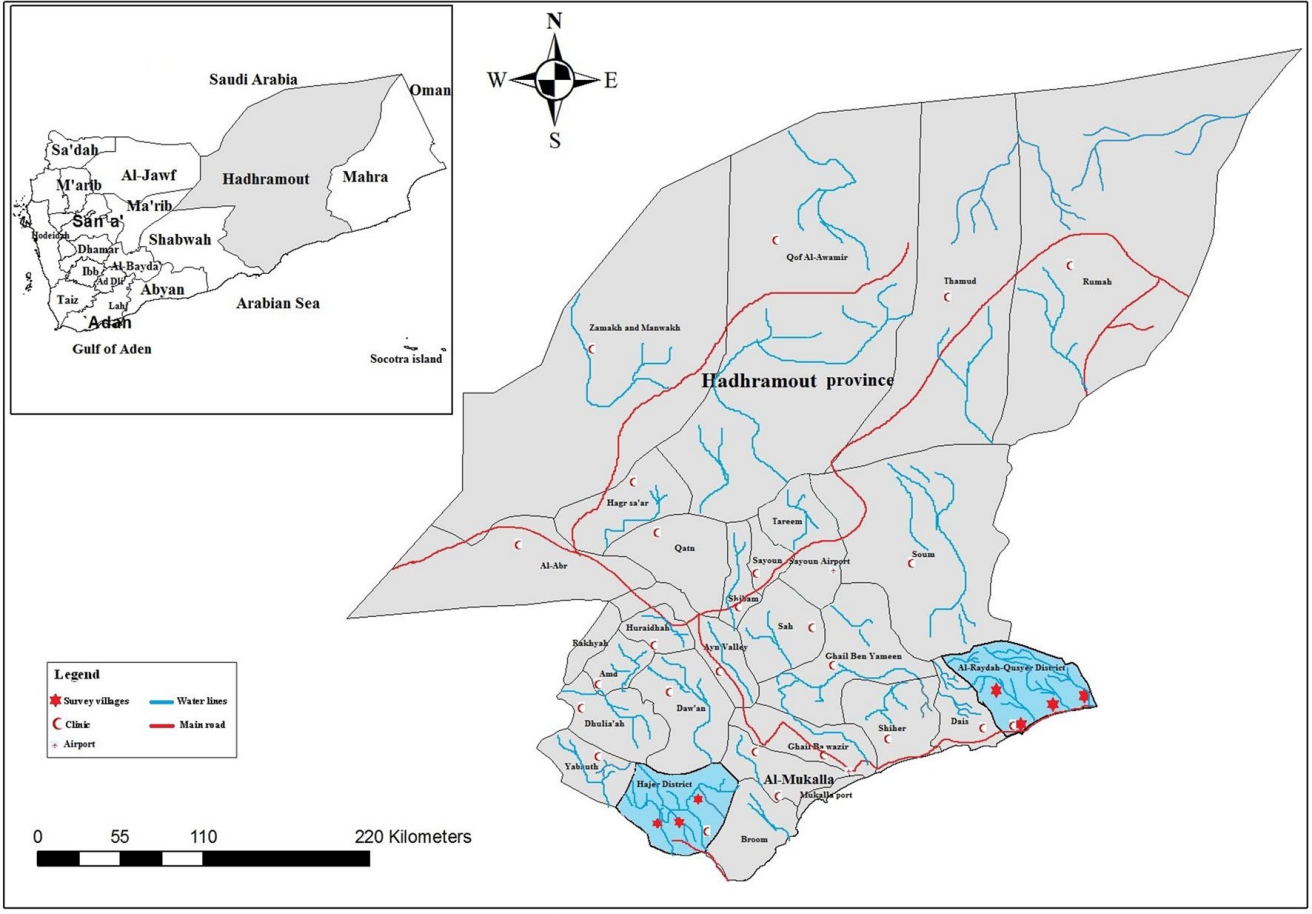
Map indicates the study area [56]

Blood samples were collected from two districts in Hadhramout governorate (Hajer and Al-Raydah–Qusyer districts) in southeastern Yemen, representing about 36 % of the total area of Yemen with an estimated population of 1,028,556 [13]. Hadhramout has a humid and hot climate which is characterized by humidity levels ranging from 18 to 93 % and temperature ranging from 18 to 38 °C. Malaria is endemic in Hajer and Al-Raydah–Qusyer districts with more than 99 % of cases caused by *P. falciparum* and few cases of *Plasmodium vivax* [2]. House-to-house survey was conducted during the transmission season from July 2011 to May 2012. Finger-prick blood samples were collected from 735 participants in three villages in Hajer and four villages in Al-Raydah–Qusyer districts (Fig. 1). These villages were selected because they are endemic malaria areas, and houses were selected randomly. Informed consent was obtained from each participant, and consent was obtained from the parents of children after the survey objectives were clearly explained to the subjects. The Faculty of Medicine, Hadhramout University for Science and Technology, and the Ministry of Health and Population, Yemen approved the study protocol. Malaria positive patients were treated by NMCP following the national anti-malarial drug policy.

### Detection of *Pfdhfr* and *Pfdhps* mutations

Archive blood spots were collected on Whatman filter paper 3MM (Whatman International Ltd., Maidstone, England) and maintained separately in clean, dry, and well-sealed plastic bag with silica gel. The bags were stored at room temperature until use. Genomic DNA was extracted from dried archive blood spots using Qiagen DNA Mini Kit for blood and tissue (QIAGEN, Germany) according to the manufacturer’s instructions. Extracted DNA was eluted using 50 µL of Qiagen AE elution buffer and stored at −20 °C until use. *Plasmodium falciparum* was identified using nested PCR based on small subunit ribosomal RNA gene [14, 15]. PCR master mix and thermal cycling conditions were performed as reported previously [16]. Genomic DNA of *Pfdhfr* gene was amplified using nested PCR following the method described previously [17, 18]. Briefly, an amplicon of 720 bp was amplified using the primers pair AMPI (5′-TTTATATTTTCTCCTTTTTA-3′) and AMP2 (5′-CATTTTATTATTCGTTTTCT-3′) in the primary PCR, and an amplicon of 700 bp was amplified using the primers SP1 (5′-ATGATGGAACAAGTCTGCGAC-3′) and SP2 (5′-ACATTTTATTATTCGTTTTC-3′) in the nested PCR. The PCR was carried out in a total of 25 μl mixture containing 1× PCR buffer, 3 mM MgCl_2_, 0.2 mM of dNTPs, 200 nM of each primer, 1 U of Taq polymerase and 4 μl of genomic DNA. Cycling condition was as follows; initial denaturation at 94 °C for 3 min, followed by 45 cycles of denaturing for 30 s at 94 °C, annealing for 1 min at 43.5 °C and extension at 72 °C for 45 s, and final extension at 72 °C for 5 min. The cycling condition for nested PCR was the same except that annealing was at 55 °C for 45 s and extension at 74 °C for 35 s, besides decreasing the number of cycles to 35 cycles.

An amplicon of 711 bp of *Pfdhps* gene was amplified by nested PCR using the outer primers pair; O1 (5′-GATTCTTTTTCAGATGGAGG-3′) and O2 (5′-TTCCTCATGTAATTCATCTGA-3′), and the nested primers; N1 (5′-AACCTAAACGTGCTGTTCAA-3′) and N2: (5′-AATTGTGTGATTTGTCCACAA-3′) [19]. The PCR mixture was as described above. The cycling condition for primary and secondary PCR was as follows; initial denaturation at 94 °C for 3 min followed by 25 cycles of denaturing for 1 min at 94 °C, annealing for 2 min at 52 °C and extension at 74 °C for 1 min and final extension at 74 °C for 5 min. PCR reagents and primers were obtained from iNtRON (iNtRON Biotechnology, Inc., Seoul, Republic of Korea. PCR product was analysed by electrophoresis in a 2.5 % agarose gel containing SYBER^®^ safe DNA gel stain (Invitrogen, USA) and visualized in a UV transilluminator. PCR products were purified with Presto™ 96 Well PCR Cleanup Kits and then sequenced in both directions using the inner primers in the ABI 3730xl DNA analyzer (Applied Biosystems). Mutations were detected by creating consensus sequences and comparing manually with the sequences in GenBank (GenBank accession numbers were XM_001351443 for *pfdhfr* and Z31584 for *pfdhps*) using BioEdit software [20].

### Statistical analysis

Data were analysed using the Statistical Package for Social Sciences (SPSS) version 22 (SPSS Inc., Chicago, IL, USA). The prevalence of a mutant allele or genotype was calculated as the percentage of the presence of the mutant allele or the genotype in the examined *P. falciparum* isolates. The difference between proportions was examined using Pearson Chi Square test or Fisher’s exact test wherever applicable. P value <0.05 was considered significant.

## Results

A total of 137 patients infected with *P. falciparum* based on microscopic examination of blood smear and nested PCR were included in the analysis of *Pfdhfr* mutations at codons 51, 59, 108, and 164, as well as *Pfdhps* mutations at codons 436, 437, and 540. The majority of patients (57.7 %) were aged >15 years old, and 88 % (121/137) of the patients did not use mosquito bed nets. 52 % (71/137) of the patients had no fever during the survey. The mean of hemoglobin was 9.5 ± 1 g/dl. The median of parasite densities was 960 asexual parasite/µl with interquartile range of 560–2333 asexual parasite/µl. The sex ratio was 1.7 males/females. Of the 137 *P. falciparum* isolates, genomic DNAs from 128 and 114 isolates were successfully sequenced for *Pfdhfr* and *Pdhps* genes, respectively.

Mutant alleles are presented in Table 1. *Pfdhfr* mutations were detected in 84 % (107/128) of *P. falciparum* isolates for codons 51 (**I**_**51**_) and 108 (**N**_**108**_) and in one isolate for codon 59 (R_59_). Mutation was not identified at codon 164. A single mutation in codon 437 (**G**_**437**_) in the *Pfdhps* gene was detected in 44.7 % (51/114) isolates. No significant difference in the distribution of the mutant alleles between Hajer and Al-Raydah–Qusyer districts was observed.

**Table 1:** Prevalence of mutant alleles of *pfdhfr* and *pfdhps* in *P. falciparum* isolates from Hadhramout, Yemen

Mutant alleles^a^	Prevalence, n (%)		Total n = 128	P value
	Hajer n = 26	Al-Raydah-Qusyer n = 102		
*Pfdhfr*				
51**I**	19 (73.1)	88 (86.3)	107 (84)	0.105
59**R**	0 (00)	1 (1.0)	1 (0.8)	0.797^b^
108**N**	19 (73.1)	88 (86.3)	107 (84)	0.105
164**L**	0 (00)	0 (00)	0 (00)	NA

Mutant alleles^a^	Prevalence, n (%)		Total n = 114	P value
	Hajer n = 25	Al-Raydah-Qusyer n = 89		
*Pfdhps*				
436**A**	0 (00)	0 (00)	0 (00)	NA
437**G**	9 (36)	42 (47)	51 (44.7)	0.56
540**E**	0 (00)	0 (00)	0 (00)	NA

*n* sample size, *NA* not applicable

^a^Mutant alleles are bold and underlined

^b^The difference was examined using Fisher exact test

Genotyping analysis based on sequences for *Pfdhfr*, *Pfdhps*, and combined *Pfdhfr*–*Pfdhps* genes was conducted. Double (**I**_**51**_C_59_**N**_**108**_I_164_) and triple (**I**_**51**_**R**_**59**_**N**_**108**_I_164_) mutant genotypes of *Pfdhfr* were detected in 82.8 % (106/128) isolates and one isolate (0.8 %), respectively. For *Pfdhps*, single mutant genotype (S_43**6**_**G**_**437**_K_540_) was detected in 44.7 % (51/114) of the isolates. Genotyping of 106 *P. falciparum* isolates for the combined *Pfdhfr*–*Pfdhps* genes showed that five (4.7 %), 46 (43 %), 42 (39.3 %), and one (0.9) isolates had single, double, triple, and quadruple mutant genotypes, respectively. Although Al-Raydah–Qusyer district had higher prevalence of mutant genotypes than Hajer district, the differences were statistically not significant (Table 2).

**Table 2:** Prevalence of genotypes of *pfdhfr,* *pfdhps*, and combined *pfdhfr*–*pfdhps* genes in *P. falciparum* isolates from Hadhramout, Yemen

Gene/genotype^a^	Prevalence n (%)		Total n = 128	P value
	Hajer n = 26	Al-Raydah-Qusyer n = 102		
*Pfdhfr*				
N_51_C_59_S_108_I_164_	7 (26.9)	14 (13.7)	21 (17)	0.105
**I**_51_C_59_ **N**_108_I_164_	19 (73.1)	87 (85.3)	106 (82.8)	0.140
**I**_51_ **R**_59_ **N**_108_I_164_	0 (0)	1 (1)	1 (0.8)	0.797^b^

Gene/genotype^a^	Prevalence n (%)		Total n = 114	P value
	Hajer n = 25	Al-Raydah-Qusyer n = 89		
*Pfdhps*				
S_436_A_437_K_540_	16 (64)	47 (52.8)	63 (55.3)	
S_436_ **G**_437_K_540_	9 (36)	42 (47.2)	51 (44.7)	0.56

Gene/genotype^a^	Prevalence n (%)		Total n = 107	P value
	Hajer n = 25	Al-Raydah-Qusyer n = 82		
*Pfdhfr*–*pfdhps*				
N_51_C_59_S_108_I_164_-S_436_A_437_K_540_	5 (20)	8 (9.8)	13 (12.1)	0.170
N_51_C_59_S_108_I_164_-S_436_ **G**_437_K_540_	1 (4)	4 (4.8)	5 (4.7)	1.000^b^
**I**_51_C_59_ **N**_108_I_164_-S_436_A_437_K_540_	11 (44)	35 (42.7)	46 (43)	0.907
**I**_51_C_59_ **N**_108_I_164_-S_436_ **G**_437_K_540_	8 (32)	34 (41.5)	42 (39.3)	0.396
**I**_51_ **R**_59_ **N**_108_I_164_-S_436_ **G**_437_K_540_	0 (00)	1 (1.2)	1 (0.9)	1.000^b^

*n* sample size, *NA* not applicable

^a^Mutant alleles are bold and underlined

^b^The difference was examined using Fisher exact test

## Discussion

This study was conducted to investigate mutations in *Pfdhfr* and *Pfdhps* genes as predictors of resistance of SP anti-malarial treatment. High prevalence (84 %) of *Pfdhfr* mutant alleles **I**_**51**_ and **N**_**108**_ was found among *P. falciparum* population in Hadhramout. These findings were higher than those from previous reports from western governorates of Yemen [11, 12]. *Pfdhfr* mutant allele **R**_**59**_ was detected in one isolate of *P. falciparum* in this study. However, a study conducted in Lahj governorate reported four samples harboring this mutant allele in 99 *P. falciparum* isolates [10]. Mutation at codon 437 of *Pfdhps* (**G**_**437**_) was also detected for the first time in 44.7 % of the examined isolates. Increased frequency of mutant alleles of *Pfdhfr* gene and emergence of new mutant alleles of *Pfdhps* gene in Yemen are early alarming signals of the possibility of decreasing in the efficacy of SP. Accumulation of mutations in *Pfdhfr* gene starts at codon 108 from serine to asparagine, resulting in low levels of pyrimethamine resistance followed by mutations **I**_**51**_ and **R**_**59**_, as well as at codon **L**_**164**_ point mutation which is related to high level of resistance [21]. Similarly, sulfadoxine resistance is induced by mutations in the *Pdhps* gene at codons 436, 437, 540, 581, and 613, that starts initially with mutation at codon 437 from alanine to glycine, followed by **E**_**540**_ and **G**_**581**_, as well as other mutations [22–24]. Emergence of resistant parasite to anti-malarial drugs involves many factors, such as economic effects, human hosts, drug pattern interactions, characteristics of the drug itself, parasites, vectors, and environmental factors [25–29].

Drug pressure could have driven the emergence and spreading of the mutant genotypes in this study. SP had been used as the second-line monotherapy for treating uncomplicated malaria for approximately more than 5 years before the introduction of ACT drug policy in 2005 [3, 5], which theoretically terminated the use of SP monotherapy. Moreover, SP is not used for intermittent preventive treatment in pregnant women in Yemen. However, SP is still available in the private sector where private physicians have poorer knowledge about the new drug policy [7, 30] emphasizing the possibility of continued use of SP monotherapy, which may result in the development of SP resistance [31]. Another possible reason could be the intensity of transmission; Hadhramout has been classified as low malaria transmission area and the initiation of pre-elimination phase was suggested [3]. The development and spreading of anti-malarial drug resistance in low transmission area has been well documented [32]. Most patients in low transmission area are usually symptomatic and receive anti-malarial treatment, which increases the chance of selecting the resistant parasite. Nevertheless, this classification is not supported by recent studies that have reported high prevalence of malaria in the community setting [2] and among asymptomatic blood donors in Hadhramout [33].

The present study showed high frequency of double mutant genotype (**I**_51_C_59_**N**_108_I_164_) among *P. falciparum* isolates. This genotype has been reported in Sudan [34, 35], Saudi Arabia [36], Angola [37], Uganda [38], Gabon [39], Iran [40] and Afghanistan [41]. *In vitro* studies showed a strong association between the *Pfdhfr* double mutant (**I**_**51**_ and **N**_**108**_) and pyrimethamine resistance in Kolkata, West Bengal of India, and Purulia [42, 43]. Another study conducted among Colombian children indicated that double mutant (**I**_**51**_ and **N**_**108**_) is significantly associated with delayed parasite clearance and plays a role in gametogenesis [44]. By contrast, a study in Sudan reported that the presence of *Pfdhfr* double mutant **I**_**51**_ and **N**_**108**_ alone is insufficient to induce in vivo resistance [45]. In this study, *Pfdhfr* triple mutant genotype (**I**_51_**R**_59_**N**_108_) was detected in one *P. falciparum* isolate. This genotype has been strongly associated with in vitro and in vivo SP resistance [46]. Mutant genotype (**I**_51_C_59_**N**_108_I_164_-S_436_**G**_437_K_540_), which combined *Pfdhfr* double mutants (**I**_51_, **N**_**108**_) and *Pfdhps* single mutant (**G**_437_), was highly prevalent among *P. falciparum* isolates. Lower frequencies of this mutant genotype compared with the present study have been reported from southeastern Iran at 2.7 % during 2008–2005 [40] and again at 1.8 % during 2008–2010 [47], as well as in Tanzania at 0.1 % [48]. Literature review showed that this genotype is not widely distributed and has not been correlated yet with the efficacy of SP either in vitro or in vivo. In this study, one isolate of *P. falciparum* harboured quadruple mutant genotype combining the triple *Pdhfr* mutant (**I**_51_**R**_59_**N**_108_) and single *Pdhps* mutant **G**_437_. Significant association between SP resistance and this genotype has been reported from in vivo studies conducted in Mali and Ghana only after 1 year of implementation of intermittent preventive treatment of malaria in infant [49, 50]. Low occurrence of this genotype has been reported from Northern Benin [51], contrary to the high prevalence reported from Southern Benin [52], Ethiopia, [53], and Senegal [54].

Anti-malarial drug policy has been designed to combine AS with longer half-live partner drug which clears the remaining parasite and prevent or delay the emergence of resistance to AS [55]. In Yemen, SP has been the partner drug combined with AS for treating uncomplicated *falciparum* malaria [3] therefore the emergence of SP resistance will expose the parasite to AS monotherapy, which has the potential to contribute to the emergence of ACT resistance in this country. In 2004, three in vivo clinical efficacy trails showed that SP monotherapy was highly efficacies for treating falciparum malaria [3]. From the time when anti-malarial drug policy had shifted from SP monotherapy as second-line to AS + SP as first-line for treating uncomplicated malaria, all in vivo efficacy trials have assessed the drug combination (AS + SP) as still being effective [8]. However, the inability of the routine therapeutic trails to distinguish between the efficacy of AS and its partner drug put SP efficacy under uncertainty particularly with the high prevalence of the double mutant genotype, which has good correlation with decreasing SP efficacy [43, 44]. In contrast, the non-emergence of quadruple *Pdhfr* mutant and triple *Pdhps* mutant genotypes that have been associated with the severe failure of SP [9] indicates that SP still provides good therapeutic response.

## Conclusion

The present study reported high prevalence of *Pfdhfr* double mutant genotype (**I**_51_**R**_59_**N**_108_) and triple *Pfdhfr*-*Pfdhps* mutant genotype (**I**_51_C_59_**N**_108_I_164_-S_436_**G**_437_K_540_) in *P. falciparum* population in Hadhramout, Yemen. These results highlight the risk of developing resistance for SP, the partner drug of AS, which subsequently will expose the parasite to AS monotherapy increasing the potential of the emergence of AS resistance in Yemen. Study findings necessitate continuous monitoring of the efficacy of the national anti-malarial drug policy in Yemen using the in vivo efficacy trails. In addition, monitoring the SP efficacy using molecular markers is crucial for early alarming of the risk of emerging AS resistance.
